# Purification and characterization of soluble recombinant Crimean-Congo hemorrhagic fever virus glycoprotein Gc expressed in mammalian 293F cells

**DOI:** 10.1186/s12896-024-00885-y

**Published:** 2024-08-27

**Authors:** Nigel Aminake Makoah, Matefo Millicent Litabe, Fredy Brice Nemg Simo, Katlego Keith Maboho, Felicity Jane Burt

**Affiliations:** 1 Division of Virology, School of Pathology, Faculty of Health Sciences, University of the Free State, Bloemfontein, 9301 South Africa; 2 Division of Virology, National Health Laboratory Service, Bloemfontein, 9301 South Africa

**Keywords:** CCHFV, Glycoproteins, Expression, Purification, Diagnostic, Chromatography

## Abstract

**Background:**

Crimean-Congo hemorrhagic fever (CCHF) is a tick-borne zoonotic disease that presents with severe hemorrhagic manifestations and is associated with significant fatality rates. The causative agent, Crimean-Congo Hemorrhagic Fever Virus (CCHFV), is a high-priority pathogen identified by the World Health Organization with no approved vaccine or specific treatment available. In addition, there is a critical need for enhanced diagnostic tools to improve public health awareness, prevention measures, and disease control strategies.

**Methods:**

We designed plasmids to enable the purification of soluble CCHFV glycoprotein Gc expressed in mammalian 293 F cells, followed by purification using affinity and size exclusion chromatography. The purified antigen was analyzed by SDS-PAGE and Western blotting to confirm its reactivity to antibodies from CCHF survivors. Additionally, an in-house indirect ELISA was developed using the purified Gc as a coating antigen.

**Results:**

The optimized expression system successfully produced soluble and pure Gc antigen after affinity chromatography. The protein showed specific reactivity with CCHFV-positive serum antibodies in Western blot analysis. The indirect ELISA assay demonstrated high efficacy in distinguishing between CCHFV-positive and -negative serum samples, indicating its potential as a valuable diagnostic tool. Size exclusion chromatography further confirmed the presence of aggregates in our protein preparation.

**Conclusions:**

The purified Gc antigen shows promise for developing direct diagnostic assays for CCHFV. The antigen’s suitability for subunit vaccine development and its application as bait for monoclonal antibody isolation from survivors could be investigated further. This work lays the foundation for future research into the development of rapid diagnostic tests for field deployment.

**Supplementary Information:**

The online version contains supplementary material available at 10.1186/s12896-024-00885-y.

## Introduction

Crimean-Congo hemorrhagic fever (CCHF) is a zoonotic disease transmitted primarily by infected ticks belonging to the genus *Hyalomma*, or through contact with contaminated bodily fluids. This disease is characterized by severe and often fatal hemorrhagic symptoms in humans and has a fatality rate that ranges from 5 to 30% but has been reported to exceed 30% in certain regions [1–3]. CCHF is endemic in Africa, Asia, the Middle East, the Balkans, and regions of eastern and southern Europe, where it causes sporadic outbreaks [4]. Misdiagnosis or underdiagnosis is common in low resource countries that lack specialised diagnostic facilities, leading to poorly defined disease prevalence [5]. Given the risk of the virus spreading to non-endemic regions, enhancing diagnostic and capacities and establishing serological surveillance programs are critical [6].

The causative agent of CCHF is the Crimean-Congo hemorrhagic fever orthonairovirus (CCHFV), a highly infectious virus that belongs to the genus Orthonairovirus within the family *Nairoviridae* and the order *Bunyavirales*. CCHFV is an enveloped virus with tri-segmented negative-sense RNA genome comprising, small (S), medium (M), and large (L) segments [7, 8]. The S segment encodes the nucleocapsid protein (NP), essential in packaging the viral RNA genome. The L segment encodes the RNA-dependent RNA polymerase, responsible for replicating the viral genome and transcribing viral RNA. The M segment encodes the glycoprotein precursor complex (GPC), which is post-translationally cleaved for the production of mature glycoproteins, Gn and Gc. In addition, it produces a GP160/85 protein that undergoes further proteolytic processing, resulting in a heavily glycosylated mucin-like domain (MLD) and a GP38 protein. The M segment also encodes a medium non-structural protein (NSm), which promotes glycoprotein processing and virion assembly. The proteolytic processing of the GPC occurs as the proteins traffic through the endoplasmic reticulum and Golgi apparatus and involves host furin-like and SKI-1 proteases [9–11].

Although commercial serological assays for diagnosis and surveillance are available, their cost is prohibitive for low-resource settings, and no rapid assays have been approved due to a lack of suitable reagents. The glycoproteins Gn and Gc are particularly valuable as antigens for the development of various immunoassays due to their role in virus-host interactions, making them prime targets for neutralizing antibodies and vaccine development [12–20]. Furthermore, Gc has been used as a bait to isolate Gc specific B cells from individuals who survived CCHF, leading to the production of monoclonal antibodies targeting the Gc, which show promise for prophylactic and therapeutic interventions [21].

While molecular and serological approaches can diagnose symptomatic patients, both may require expensive equipment or a biosafety level (BSL) 4 facility. Therefore, enhancing serological assays for surveillance remains critical to track the virus’s spread and providing affordable diagnostic alternatives. One of the challenges in achieving these objectives has been the difficulty in producing soluble CCHFV glycoproteins. The expression of soluble recombinant glycoprotein Gc has previously been achieved in Drosophila Schneider 2 (S2) cells after truncating 17 amino acids from the C-terminus of the Gc ectodomain [22]. Furthermore, a construct with an internal deletion of residues 520–1039, fusing Gc directly to the MLD and GP38 and expressing the fusion proteins in stably transfected S2 cells and the Gc protein was secreted and harvested from clarified S2 culture supernatant via a C-terminal double strep-tag II sequence using affinity chromatography for further analysis [21]. Recent studies have solved the structure of CCHFV Gc using X-ray diffraction and cryo-electron microscopy [23, 24].

Expression systems for recombinant proteins using mammalian cells can introduce correct protein folding and post-translational modifications, which are often essential for full biological activity [25]. A recombinant CCHFV glycoprotein Gc produced in HEK293 cells and purified from culture supernatant is currently commercialized by the Native Antigen company and has recently been used for the development of an in-house ELISA [26]. However, detailed reports on the design and production of this essential protein remain limited.

In this study, we report for the first time the design and purification of soluble recombinant CCHFV Gc antigen from the South African strain SPU187/90 [27], expressed in 293 F cells using the human insulin leader peptide. We also investigate CCHFV Gc antigenic property and give an insight into the potential quaternary structure of the expressed glycoprotein.

## Materials and methods

### Human samples

All the participants provided written informed consent prior to the study. The study was approved by the University of Free State’s Health Sciences Research Ethics Committee. A total of 12 serum samples from previously laboratory confirmed CCHFV-infected individuals and 11 CCHFV-negative control samples, isolated from volunteers with no previous history of CCHF virus infection were used in this study. CCHF positive and negative samples were previously tested using commercial Crimean-Congo fever virus Mosaic 2 (IgG) kit (Euroimmun, Germany) which contains slides for use in the IFA [28]. Each slide contains ten fields, and each field contains three BIOCHIPS, coated with non-transfected cells, cells expressing the CCHFV NP and cells expressing the CCHFV Gc. The 12 positive samples used in this study were collected between 2008 and 2011 (Ethics reference number: HSREC 95/2016) and the 11 negative samples were collected in 2013 (Ethics reference number: HSREC 95/2016). All samples were heat inactivated at 54ºC for 30 min before storing at -80 ºC.

### Cells

For the transfection experiments, adherent HEK-293 cells (ATCC CRL1573, ATCC, USA) were specifically used for immunofluorescence assays (IFA) and were grown in Dulbecco’s Modified Eagle Medium (DMEM) supplemented with 5% gamma-irradiated fetal bovine serum (ThermoFisher Scientific, UK), 1% L-glutamine (Lonza, Belgium), 1% penicillin-streptomycin (Lonza, Belgium) and 1% non-essential amino acids (Lonza, Belgium) and cultivated at 37⁰C, 5%CO_2_. The 293 F (Obtained from Prof. Penny Moore) cells were utilized for protein expression and purification and were cultivated in Freestyle™ 293 Expression medium (ThermoFisher Scientific, UK) and incubated at 37⁰C, 8% CO_2_, with shaking in a Brunswick™ S41i incubator (Eppendorf, Germany).

### Construct design and cloning

The mammalian cell codon-optimized nucleotide sequence coding for the glycoprotein of the CCHFV isolate SPU187/90 (Genbank: KJ682814.1) was synthesized commercially (GenScript, USA), the strain was isolated from an abattoir worker in the northwest province in South Africa, who tragically succumbed to a fatal CCHF disease [27]. Primers were designed to amplify the Gc ectodomain (from amino acid 1050 to 1594 of SPU 187/90), and a C-terminally truncated version (from amino acid 1050 to 1577). The signal peptide of the CCHFV glycoprotein (GPC: MHISLMYAVLCLQLCSLG) or the human insulin leader sequence (INS: MALWMRLLPLLALLALWGPDPAAA) were inserted in the forward primers together with a Kozak sequence and the EcoRI restriction site, while an Avi-tag (GLNDIFEAQKIEWHE), a hexahistidine tag and the XhoI restriction site were inserted in the reverse primers (Table 1). The Gc gene was amplified from a plasmid containing a codon optimized SPU187/90 M-segment, using Phusion PCR polymerase following conditions described in Table 1 and cloned into the mammalian expression vector pCDNA3.1(+) using EcoRI and XhoI restriction sites. Ligation reactions were used to transform beta-10 competent cells (New England Biolabs, USA) and positive transformants were identified using Sanger sequencing.

**Table 1:** Primers used to generate expression constructs and PCR conditions

Constructs	Primer names	Sequences	number of bases	cloning PCR conditions	PCR product
Construct 1	Fwd-2-Gc	GCATCTAGA*GAATTC*GCCACCATGCATATTAGCCTGATGTATGCGGTGCTGTGCCTGCAGCTGTGCAGCCTGGGCTTCCTGGACAGCATCGTG	93	• Initial temp: 98° C, for 30 s. 35 cycles of: • denaturation at 98° C for 10 s, • annealing at 72° C for 30 s, and • extension at 72° C for 1 min. and 30 s.	Gc ectodomain with GPC leader, Avi-tag and His-tag
	Rev-2-Gc	GCA*CTCGAG*TTAGTGGTGGTGGTGGTGGTGCTCGTGCCACTCGATCTTCTGGGCCTCGAAGATATCGTTCAGGCCCACGTTGCCGAAGATGCC	93		
Construct 2	CCHFV-Gc-F_Ins	GCATCTAGA*GAATTC*GCCACCATGGCCCTGTGGATGAGACTGCTGCCTCTGCTGGCCCTGCTCGCCCTGTGGGGCCCTGACCCCGCCGCCGCTTTCCTGGACAGCATCGTG	111	• Initial temp: 98° C, for 30 s. 35 cycles of: • denaturation at 98° C for 10 s, • annealing at 72° C for 30 s, and • extension at 72° C for 1 min. and 30 s.	Gc ectodomain with human insulin leader, Avi-tag and His-tag
	Rev-2-Gc	GCA*CTCGAG*TTAGTGGTGGTGGTGGTGGTGCTCGTGCCACTCGATCTTCTGGGCCTCGAAGATATCGTTCAGGCCCACGTTGCCGAAGATGCC	93		
Construct 3	CCHFV-Gc-F_Ins	GCATCTAGA*GAATTC*GCCACCATGGCCCTGTGGATGAGACTGCTGCCTCTGCTGGCCCTGCTCGCCCTGTGGGGCCCTGACCCCGCCGCCGCTTTCCTGGACAGCATCGTG	111	• Initial temp: 98° C, for 30 s. 35 cycles of: • denaturation at 98° C for 10 s, • annealing at 72° C for 30 s, and • extension at 72° C for 1 min. and 30 s.	Gc ectodomain with truncated C-terminal, human insulin leader, Avi-tag and His-tag
	CCHF-Gc-R (-51)	GCA*CTCGAG*TTAGTGGTGGTGGTGGTGGTGCTCGTGCCACTCGATCTTCTGGGCCTCGAAGATATCGTTCAGGCCCTTCACGCTCTCCAGCCAGCAG	97		

### Immunofluorescence assay

To confirm the expression of Gc from construct 1, an IFA was carried out on transfected HEK293. Transfected cells were detached using trypsin (#TRY-4B; ThermoFisher Scientific, UK) and centrifuged for 5 min at 500 g, the supernatant was discarded, and cells were re-suspended in PBS (#10010015ThermoFisher Scientific, UK). 10 µl aliquot of the cells was added to each well of the 10 well slides (#X2XER208B; New Erie Scientific, USA) and left to air-dry for an hour, then fixed with methanol and acetone in a 1:1 ratio for 30 min at minus 20 °C. An IgG CCHF positive serum sample diluted 1:100 in blocking buffer (1% BSA in PBS) was applied to each well and incubated for 30 min at 37 °C. A CCHF negative serum was used as control. The IFA slides were washed three times for one minute with PBS to remove unbound antibodies. The secondary antibody, goat anti-human IgG conjugated to fluorescein isothiocyanate (#172–1006; KPL, USA), diluted 1:20 in 0.1% Evans blue (#2589375; Merck, Germany) was then added and incubated at 37 °C for 30 min. The slides were washed again three times with PBS for one minute and were left to air dry. The slides were then mounted by inverting the slides onto mounting media (#F180907DO; Euroimmun, Germany) on a cover slip (#0101142; Marienfeld, Germany). Cells were observed for fluorescence using a Nikon Eclipse Ni fluorescent microscope (Nikon, Japan), and the image of fluorescent cells was captured with the mounted Camera at 40x magnification.

### Expression and purification of recombinant CCHFV Gc

Recombinant proteins were produced by transfecting 293 F cells with purified plasmid DNA using PEI-max (#A803669; Polysciences, USA). Briefly, 293 F cells were cultivated until concentration reached 2 × 10^6^ cells/ml. DNA was mixed with PEI-max at a 1:3 mass ratio (w/w) and incubated a room temperature for 20 min. The mixture was then added to cells and incubated at room temperature for 20 min, then transferred in the shaking incubator and incubated at 37⁰C, 8%CO_2_. After 72 h incubation the supernatant from transfected cells was harvested and centrifuged at 4,000 g for 30 min. The supernatant was then passed through a Nickel-NTA Agarose (#88222; ThermoFisher Scientific, USA) column and allowed to flow by gravity. The column was then washed with a washing buffer (50 mM Tris-HCl, pH8.0, 20 mM imidazole, 300 mM NaCl) and the recombinant CCHFV Gc, bound to the column was eluted with elution buffer (50 mM Tris-HCl, pH 8.0, 300 mM Imidazole, 150 mM NaCl). The protein was concentrated using a 30 KDa cut-off Amicon centrifugal units (#UFC903024;Merck, Germany) and proteins were stored in PBS, pH7.4. Size-exclusion chromatography (SEC) was further performed with a Superdex 200 (10/300) GL column (#28990944; Cytiva, USA) equilibrated at a flow rate of 0.3 mL/min using an AKTA Go^™^ protein purification system (Cytiva, USA). Purified Gc and gel filtration standard (#1511901; Biorad, USA) were eluted at a flow rate of 0.5 mL/min.

### SDS-PAGE and western blotting

The purified recombinant CCHFV Gc was analysed by SDS-PAGE and Western blotting. To determine protein yield and purity, samples were mixed with 2x Laemmli sample buffer (non-reducing) or 5x lane marker reducing sample buffer (#; ThermoFisher Scientific, UK) and loaded onto a NuPAGE™ 4–12% Bis-Tris gel (#NP0322PK2;Invitrogen, USA) and proteins were visualized with GelCode™ Blue Safe protein (#24590;ThermoFisher Scientific, UK). Protein concentrations were estimated using Nanodrop™ (Thermofisher Scientific, UK) at 280 nm. For the western blot analysis, the eluted protein was first separated on a SDS-PAGE, then transferred onto a nitrocellulose membrane using a transblot turbo starter system (Biorad, USA) and the membrane was incubated with anti-CCHFV serum sample as primary antibody (dilution 1:200), then detected using goat anti-human IgG alkaline phosphatase (AP)-labelled secondary antibody (dilution 1:10000; #AP124A; Merck, Germany). The expression and the target protein were then visualized by BCIP/NBT substrate (#34042ThermoFisher Scientific, UK) reaction.

### Native PAGE

The purified recombinant CCHFV Gc protein was further analyzed using a NativePAGE™ 4–16% Bis-Tris gel (#BN1002BOX; Invitrogen, USA). The CCHFV Gc was prepared by mixing with NativePAGE™ 4x sample buffer (#BN2003; Invitrogen, USA) and 1%Triton X-100 (BDH Chemicals, UK). The NativeMark™ (#;LC0725 Invitrogen, USA) unstained protein standard was resolved alongside CCHFV Gc to estimate the size. After electrophoresis, the protein and markers were stained using GelCode™ Blue Safe protein (ThermoFisher Scientific, UK) and de-stained using a de-staining solution (10% Acetic acid and 50% methanol diluted in distilled water).

### Anti-CCHFV Gc-specific ELISA

Recombinant CCHFV Gc protein was used for the development of an indirect IgG ELISA. Unless stated otherwise, all volumes were 100 µl/well, plates were incubated at 37ºC for an hour, and washed three times for 15 s with PBS containing 0.1% tween 20 (#655204;Promega, USA). Briefly, 96-well PolySorp microtiter plates (#162161;Nalgene Nunc International Corporation, USA) were coated with 0.5 µg/ml of purified CCHFV Gc antigen and incubated overnight at 4ºC. The following day, plates were washed and blocked with 200 µl of 5% skim milk/PBS and incubated. Post-incubation plates were washed and incubated with 10-fold serially diluted serum in duplicates, serial dilutions were prepared in 2% skimmed milk/PBS. Samples were diluted 10-fold from 1 × 10^1^ to 1 × 10^7^. On each plate the bottom row was reacted with PBS and used as a control to calculate the net OD. Post-incubation, plates were washed and incubated with goat anti-human IgG conjugated to horseradish peroxidase (#5220 − 0361;SeraCare, USA), diluted 1:10000. Post-incubation plates were washed and visualized by adding 2,2′ -Azino di-ethyl-benzothiazoline-sulfonic acid peroxidase substrate (ABTS) (#5120-0042;SeraCare, USA). Plates were then incubated for 10 min at room temperature in the dark and the OD values were read at 405 nm using the 800™ TS microplate reader (Biotek, USA). Net OD values were determined for each sample as follows: net OD = OD values from serum samples minus OD values from 2% skimmed milk/PBS. The same protocol was used to compare the reactivity of a CCHF-positive and CCHF-negative samples on CCHFV Gc aggregates and the monomers with serum dilution starting at 1:100.

### Statistical analysis

Statistical differences between CCHF negative and CCHF positive samples were analysed using an unpaired t-test using GraphPad Prism (version 9.5.1). Statistical difference between the reactivity of a CCHF positive sample against Gc aggregates and Gc monomers was analyzed using a paired t-test. *P*-value of less than 0.05 was considered statistically significant.

### Database and Computional Biology servers

We obtained signal peptide sequences from the signal peptide database (http://www.signalpeptide.de/index.php?sess=&m=listspdb_mammalia&start=11294&orderby=id&sortdir=as) and added them at the N-terminal end of the Gc amino acid sequence. We then used SignalP 5.0 (https://services.healthtech.dtu.dk/services/SignalP-5.0/) to predict the location and the probability of the signal peptide cleavage sites in the Gc sequence. We estimated the theoretical molecular weight of CCHFV Gc using Compute pI/Mw tool on Expasy (https://web.expasy.org/compute_pi/). To determine the sequence of the ectodomain to express, we used the transmembrane predictor TMHMM 2.0 (https://services.healthtech.dtu.dk/services/TMHMM-2.0/). To predict N-glycosylation sites on the amino sequence of Gc, we used NetNGlyc – 1.0 (https://services.healthtech.dtu.dk/services/NetNGlyc-1.0/). The plasmid was designed using ApE plasmid editor (https://jorgensen.biology.utah.edu/wayned/ape/) and the map was obtained by opening the file in SnapGene 7.2 (https://www.snapgene.com/).

## Results

### Design, expressing and purification of CCHFV Gc protein

**Fig. 1:**
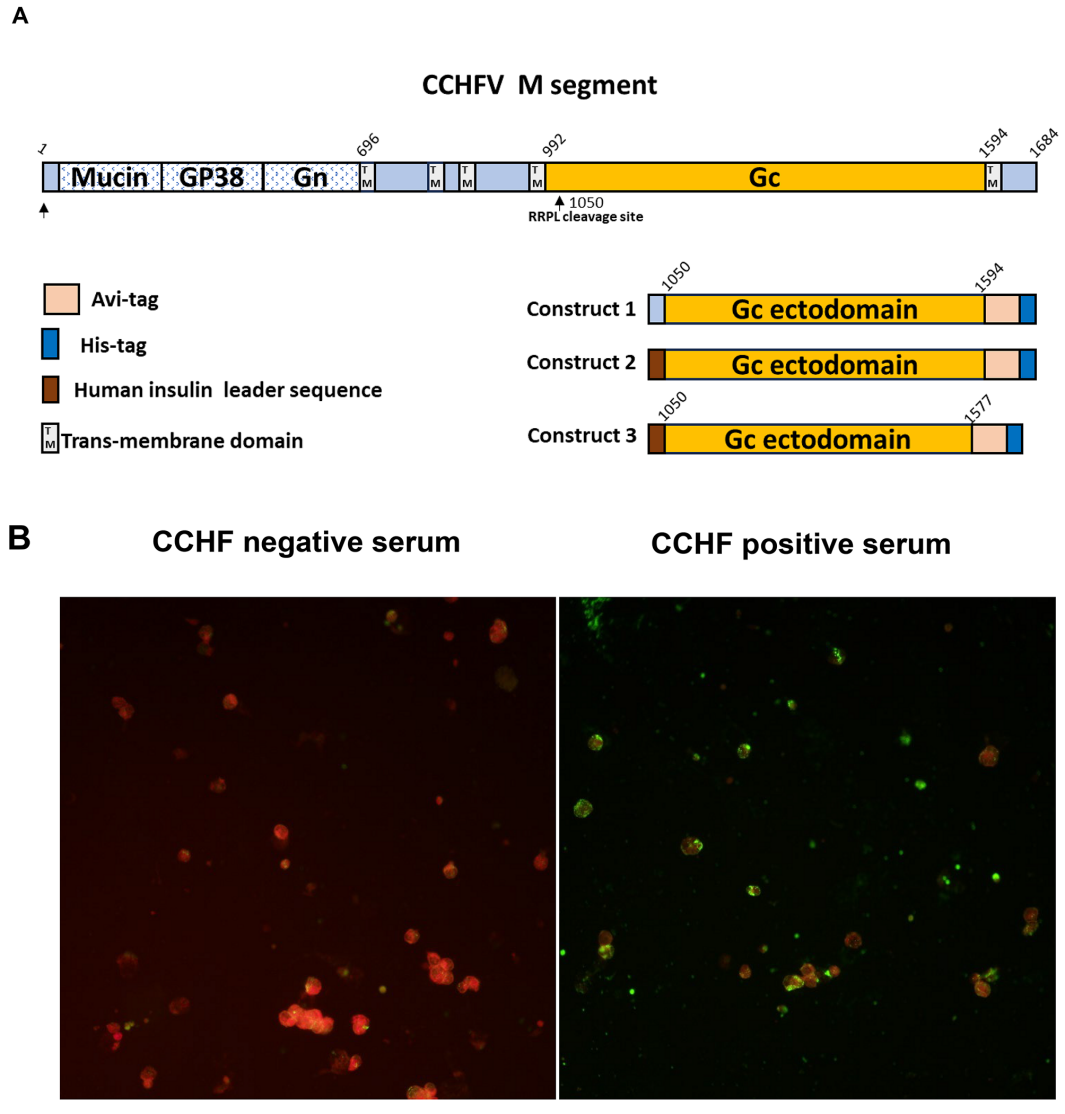
**A**. Schematic depicting the M-segment of SPU 187/90 (top) encoding the mucin-like domain GP38, Gn, and Gc and transmembrane domains. Below are the constructs for recombinant protein expression (bottom right). Numbers correspond to full-length glycoprotein amino acid precursor numbering. Ectodomains, transmembrane (TM) domains, and endodomains were predicted using the TMHMM server v. 2.0. **B**. Localization of expressed CCHFV Gc by immunofluorescence assay: HEK293 cells transfected with construct 1 were stained with the human anti-CCHFV serum sample followed by the goat anti-human IgG (H + L) antibody conjugated with FITC. A negative CCHF serum was used as a control

**Fig. 2:**
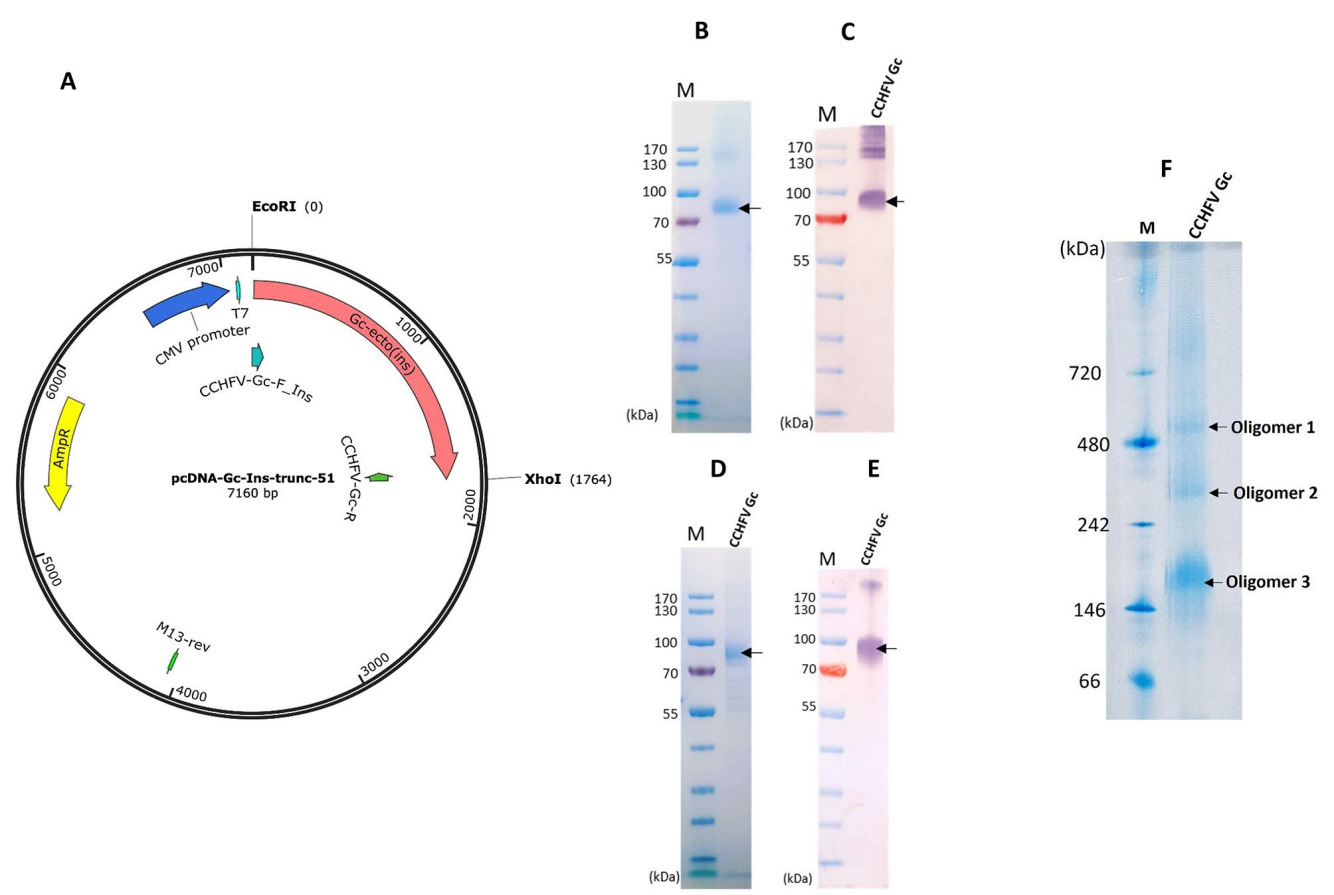
**A**. Schematic of the plasmid: Gc-ecto(Ins) represents construct 3 in Fig. 1A. **B-C**. Analysis of CCHFV Gc under non-reducing conditions: Supernatant from construct 3 transfected 293 F cells was passed through a nickel agarose column and the eluted protein was analyzed by SDS-PAGE (**B**) and western blotting **(C). D-E**: Analysis of CCHFV Gc under reducing conditions. Supernatant from construct 3 transfected 293 F cells was passed through a nickel agarose column, the eluted protein was treated with the reducing agent DTT and analyzed by SDS-PAGE (**D**) and western blotting (**E**). **F**: Analysis of CCHFV Gc by Native PAGE. Supernatant from construct 3 transfected 293 F cells was passed through a nickel agarose column, the eluted protein was treated with 1% triton X-100 and resolved on a 4–12% native PAGE gel. PageRuler™ Pestained Protein Ladder (#26616;ThermoFisher Scientific, UK) was used to estimate the size of the protein on the SDS-PAGE gels and Western blot membranes

A total of three recombinant plasmids were engineered containing CCHFV envelope protein Gc (Fig. 1A and S2). The initial construct encoded the entire ectodomain along with the GPC leader sequence for secretion. However, we only observed Gc expression via IFA (Fig. 1B) as depicted by the green fluorescence in the cellular compartment of HEK293 cells, with no detectable secretion into the culture supernatant. To achieve secretion, we searched for an optimal leader sequence by comparing signal peptide sequences from the database with GPC leader sequence using Signal P 5.0. A subsequent plasmid encoding the Gc ectodomain alongside a human insulin leader sequence, predicted to have a higher cleavage probability than the GPC leader (Fig. S1) did not result in secretion of CCHFV Gc in growth media. In our third construct, we implemented a previously described 17-amino acid truncation [22] reported to enhance solubility in insect cells. We successfully achieved secretion using the third construct (Fig. 2A), and soluble CCHFV was purified using affinity chromatography. This suggests that the 17-amino acid truncation was the primary determinant of secretion.

### CCHFV Gc analysis

In our investigation of the expressed CCHFV Gc protein, we employed SDS-PAGE with a gradient gel under non-reducing conditions, and this analysis revealed the presence of a recombinant CCHFV Gc migrating between the 70 and 100 kDa molecular weight marker (Fig. 2B). The size of CCHFV Gc on the gel is slightly larger than the theoretically expected value of 65 kDa (Fig. S2). Expression and purification of CCHFV Gc ranged from 1.3 to 1.7 mg/L of culture. Further examination through Western blot analysis using CCHFV positive serum demonstrated the specific binding of serum antibodies to the recombinant CCHFV Gc (Fig. 2C). The presence of additional bands above the CCHFV Gc protein on the SDS-PAGE and Western blot may indicate the presence of tertiary, quaternary structures, or aggregates. To assess the potential nature of the additional bands, we first repeated the analysis under reducing conditions, and results indicated that the high molecular weight proteins appeared to be reduced (Fig. 2D and E), especially the prominent bands between the molecular weight markers 130 and 170 kDa. The size of CCHFV Gc remained unchanged whether analyzed under reducing or non-reducing conditions and a high molecular weight band, above the 170 kDa persisted on the western blot under reduced condition. To get an insight into the high molecular weight structures, we further assessed CCHFV Gc using Native PAGE and SEC. The native PAGE indicated the formation of three apparent oligomeric structures of higher molecular weight. (Fig. 2F).

**Fig. 3:**
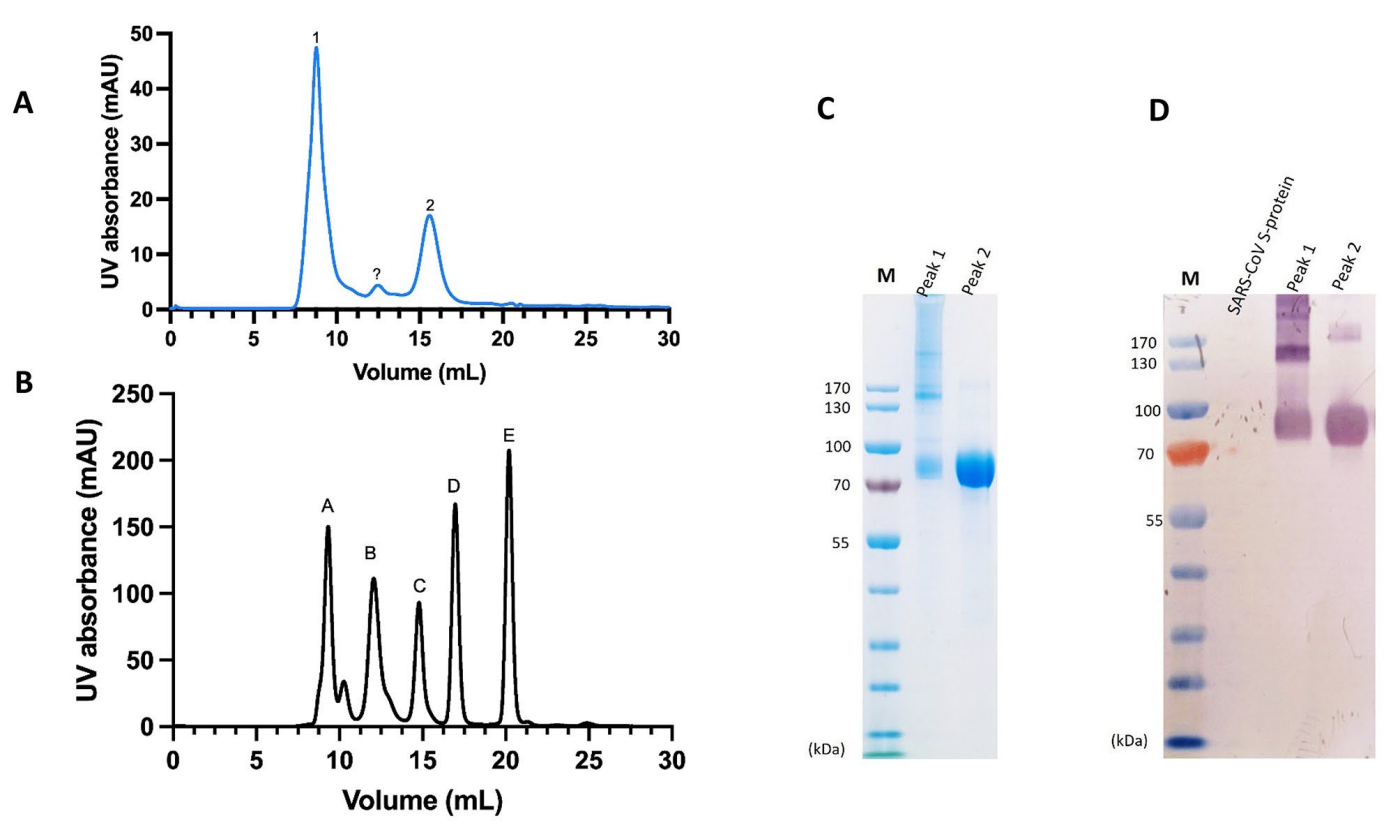
**A**. SEC elution profiles of CCHFV Gc: Gc protein purified by affinity chromatography was concentrated and stored at -80⁰C. Subsequently thawed and loaded on a AKTA™ Go and separated using a Superdex 200 increase 10/300 GL column. The stored solution is composed of aggregates (peak 1), multimers (peak?) and monomers (peak 2). **B**. SEC elution profiles of Gel filtration standard: chromatogram of Gel Filtration Standard (Bio Rad cat# 151–1901) comprised of five proteins. The profile denotes five peaks of which molecular weights can be determined. From left to right, peak A: thyroglobulin (670 kDa), peak B: gamma globulin (158 kDa), peak C: ovalbumin (44 kDa), peak D: myoglobin (17 kDa), peak E: Vitamin B12 (1.35 kDa). **C**. Analysis of CCHFV Gc under non-reducing conditions by SDS-PAGE: Approximately 4 µg of peak 1 and peak 2 were loaded on the gel and separated as described in the method. **D**. Analysis of CCHFV Gc under non-reducing conditions by Western blot: Approximately 4 µg of peak 1 and peak 2 were loaded on the gel, separated, and blotted as described in the method. The SARS-CoV S-glycoprotein tagged with Avi- and Histidine using the same approach described for CCHFV Gc was used as a control in the experiment. PageRuler™ Pestained Protein Ladder (#26616;ThermoFisher Scientific, UK) was used to estimate the size of the protein on the SDS-PAGE gels and Western blot membranes

SEC analysis of Gc revealed that there were two significant peaks (Fig. 3A and B). Peak 1 appears to be an aggregation of Gc monomers, and the other distinct peak 2 appears to be the Gc monomers. A smaller peak (peak?) between the two major peaks appears to be an oligomer of CCHFV Gc. SDS-PAGE and Western blot of peak 1 and peak 2 showed a distinct pattern. Peak 1 nearly recapitulated the Gc preparation before SEC as observed in Fig. 2B, while peak 2 is predominantly the monomeric Gc and the faint band above the 170 kDa marker observed on the gel (Fig. 3B and C) could explain the presence of the small peak (peak?) in the SEC profile. Peak 1 and Peak 2 were further analysed using native PAGE and Peak 1 contains oligomers 1 and 2 as observed in Fig. 2F while peak 2 corresponded to oligomer 3. The oligomer 3, migrated between the molecular weight marker 146 kDa and 242 kDa on the native gel as observed in Fig. 2F.

### Reactivity of recombinant CCHFV Gc to CCHF positive serum

**Fig. 4:**
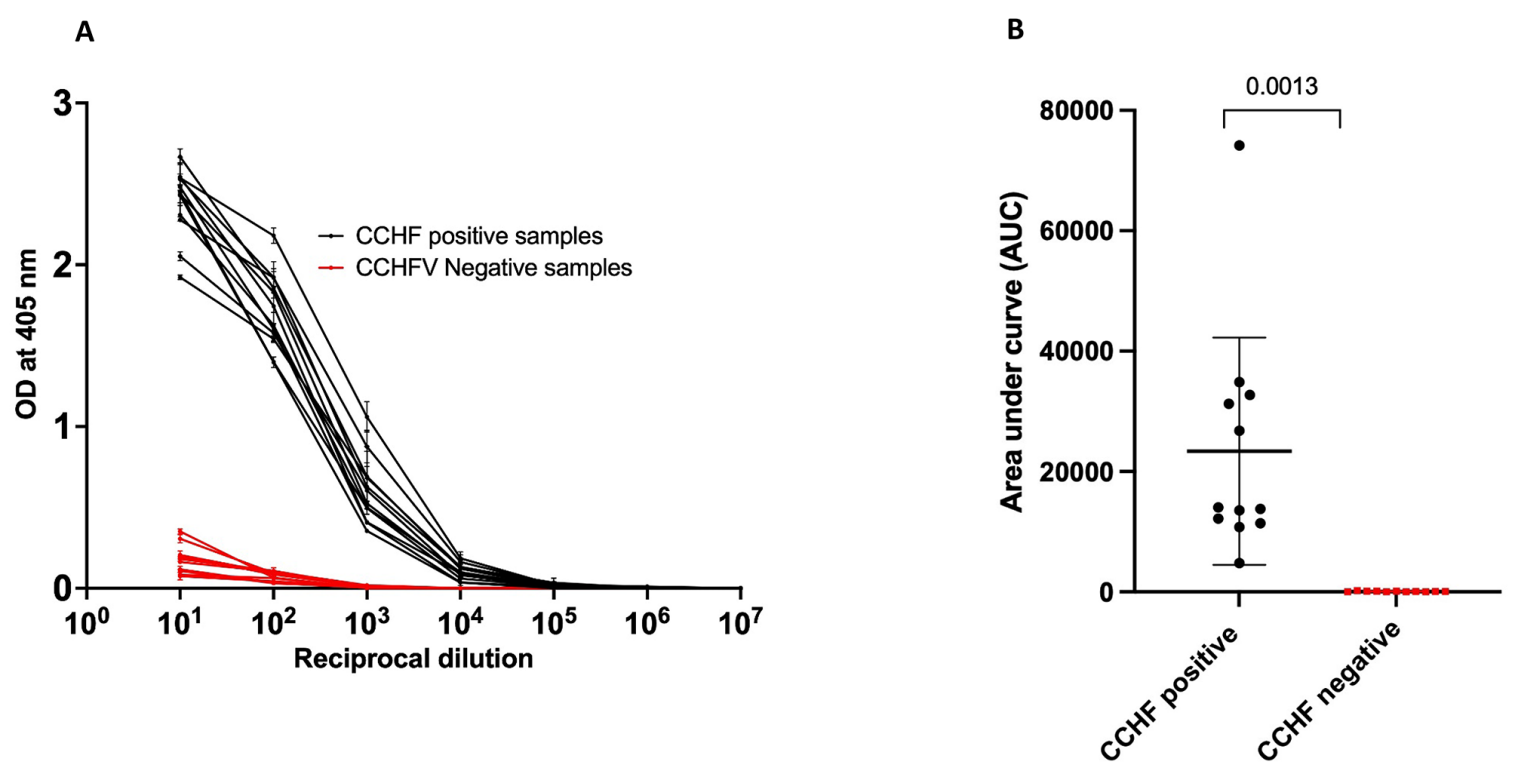
Reactivity of controls and CCHF positive sera to CCHFV Gc: **A**. ELISA curves showing sera reactivity of CCHFV Gc to CCHF-positive and CCHF-negative serum samples. **B**. Area under the curve (AUC) of positive and negative serum samples

**Fig. 5:**
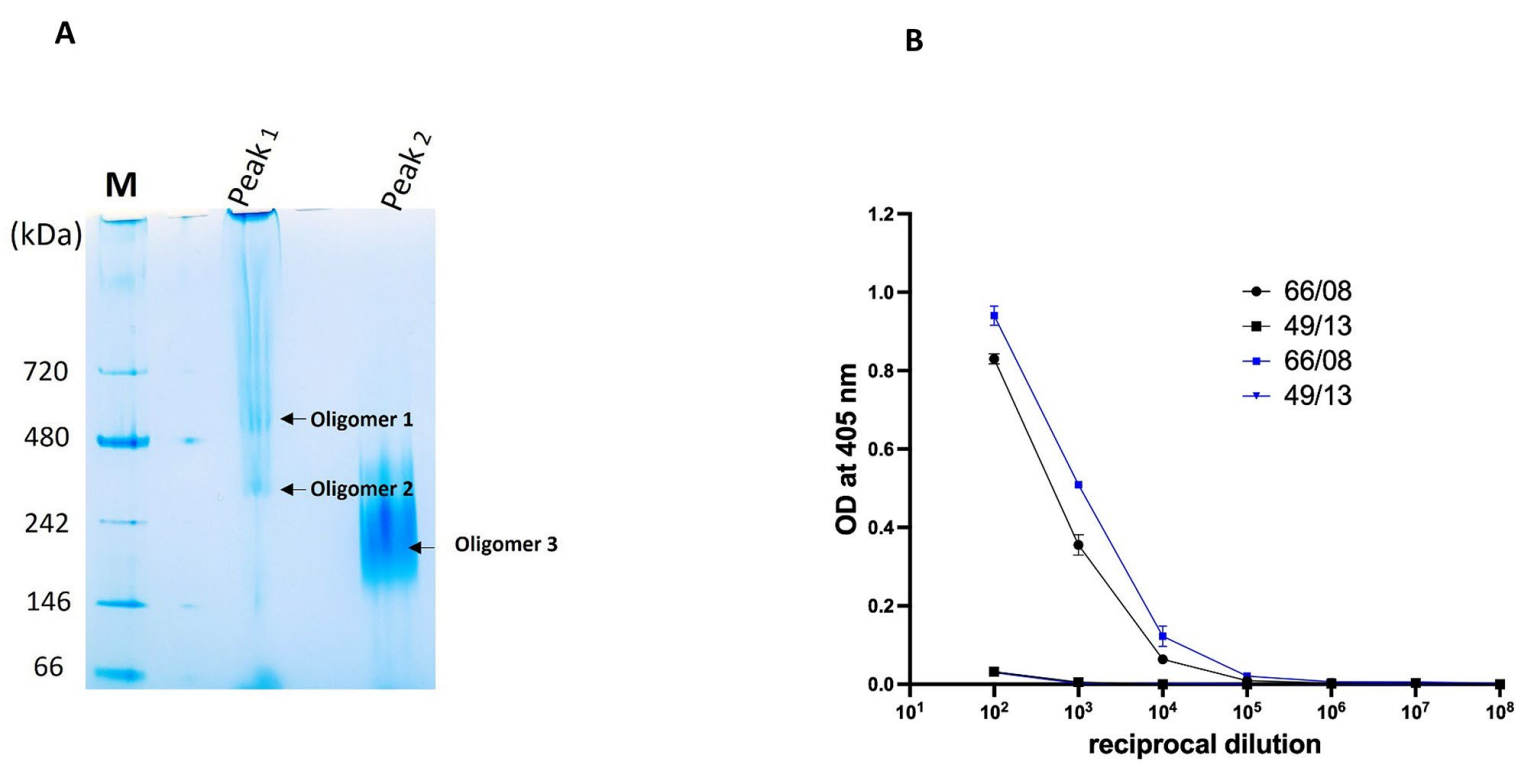
**A**. Native PAGE: Analysis of CCHFV Gc (Peak 1 and 2) by Native PAGE. Approximately 10 µg of the protein obtained from SEC was treated with 1% triton X-100 and resolved on a 4–12% native PAGE gel. **B**. ELISA: curves showing sera reactivity of CCHFV Gc monomer (blue) and aggregates (black) to CCHF-positive (66/08) and CCHF-negative (49/13) serum samples

We developed and used an in-house ELISA using the Gc protein to ascertain its accuracy and precision in the detection of IgG antibodies in human sera. To evaluate its performance, 12 positive and 11 negative serum samples were diluted tenfold from 10^1^ to 10^8^. The net OD values obtained were utilized to calculate the area under the curve (AUC) for each sample. All 12 serum samples from individuals with previously confirmed reactivity to NP and Gc using a commercial IFA [28] displayed a robust response to the CCHFV Gc recombinant protein purified in this study (Fig. 4A, table S1), confirming the detection of anti-CCHFV IgG and 100% concordance with the commercially available IFA performed previously [28]. In contrast, the OD values for the negative serum samples were consistently tenfold lower in intensity when compared to the positive samples. This stark contrast underscores the assay’s capacity to effectively differentiate between positive and negative serum specimens. Importantly, the AUC values for the CCHF positive samples were significantly higher (*p* = 0.0013) in comparison to the negative samples (Fig. 4B). We further compared the reactivity of Gc aggregates and the monomeric Gc (Fig. 5B). We use a positive and a negative sample for the comparison and we found out that both reacted to CCHFV IgG in serum and there was no significant difference between the two (*P* = 0.087). This result suggests that Gc can be used for ELISA directly after affinity chromatography with no requirement for SEC.

## Discussion

CCHFV is a pathogen transmitted by ticks covering a broad geographical range. As a result, CCHF is progressively expanding following tick migration [29, 30]. Despite its propensity to spread to non-endemic regions and cause sporadic outbreaks, no preventative treatment currently exists. There is an urgent need to develop alternative treatment options and enhance diagnostic capacity for improved disease control and case management until a prophylactic vaccine is available. The effective production and purification of the soluble Gc protein from CCHFV offer great potential for various applications in diagnostics, vaccine, and antibody-based therapies. Nevertheless, the scale-up production of this protein has presented notable difficulties.

We designed a plasmid and introduced modifications that led to the expression and purification of CCHFV Gc using an efficient one-step affinity chromatography purification with nickel agarose. This outcome demonstrates that incorporating an effective leader peptide and truncating 17 amino acids, as previously documented in insect cells [22], can enhance the solubility and secretion of CCHFV Gc in mammalian 293 F cells. The analysis of the CCHFV Gc protein through SDS-PAGE revealed a highly pure recombinant protein with a molecular weight slightly exceeding the theoretical molecular weight of 65 kDa. Most enveloped viruses contain one or more glycoproteins, usually found as oligomers embedded within the lipid membrane [31]. The correct arrangement of CCHFV Gc within the membrane has yet to be clarified [23], as well as receptors on the target cells. The soluble CCHFV Gc expressed in this study has three asparagines predicted to be N-glycosylated (Fig. S3). N-glycosylation has been documented in CCHFV glycoproteins [32–34], and the glycosylation of Gc was previously studied by removing N-linked and immature carbohydrates through digestion with PNGase F and endo-H, respectively [35]. The digested Gc migrated faster than the non-digested, indicating the presence of glycans. In this study, we did not assess glycosylation. However, data from previous studies [35] explain the increased molecular weight observed on the SDS-PAGE. Western blot analysis further confirmed the specificity of this protein, as it reacts to a human anti-CCHFV serum. The absence of IgG binding to the SARS-CoV S-glycoprotein tagged with avi-tag and histidine tag in the western blot further validates that the binding is specific to Gc and not the tags. The SDS-PAGE and western blot in the presence or absence of treatment with the reducing agent DTT (Fig. 2B, C, D and E) also show that some aggregation could result from incorrect disulfide bond formation as the molecular weight of the CCHFV Gc remained unchanged. The SEC profile (Fig. 3A and B) supports the presence of aggregates (oligomer 1 and 2) and the presence of monomeric Gc in solution as previously described [24]. The sequence of CCHFV Gc expressed in this study contains 27 cysteines, suggesting the possibility of disulphide bonds formation between CCHFV Gc monomers, or Gc and other intra- or extracellular (from media) proteins. The high molecular weight proteins observed on the Western blot between 130 and 170 kDa (Fig. 2C and E) appeared to be reduced by DTT, indicating the presence of oligomers resulting from disulfide bonds formation. When the protein sample was analysed by native PAGE, CCHFV Gc monomer in peak 2 was resolved as a single band at a molecular weight between 146 and 242 kDa (Fig. 5A), which is larger than the monomer observed between80-90 kDa on the SDS-PAGE (Fig. 3C). A similar discrepancy was previously reported when analysing CLpA for crystallization [36]. The discrepancy between the profiles observed in native PAGE and SDS-PAGE were attributed to further oligomerization of the monomeric protein during migration in the native gel. This highlights a complex behavior of certain protein under various conditions. While the potential of CCHFV Gc as a diagnostic target is well recognized, these findings highlight the need for additional studies to fully elucidate its structural properties.

We used the purified CCHFV Gc as a coating antigen in an in-house developed ELISA to detect IgG in CCHF-positive sera. This preliminary evaluation, using a small sample set previously tested by a commercial IFA, was aimed at determining whether our ELISA will replicate the results previously obtained. Our ELISA results (Fig. 4A) showed that all 12 positive samples displayed a strong signal, whereas the negative samples exhibited considerably lower optical density (OD) values. The AUC values suggest that the ELISA could effectively discriminate between positive and negative serum samples. However, the number of samples used was insufficient to validate our assay for diagnostic in humans, as this requires reliable validation panels from different endemic regions. Based on our findings, CCHFV Gc produced in this study could be used as a reliable antigen for identifying IgG antibodies in humans using serologic assays. Most importantly, lateral flow assays for point-of-care are needed in resource-limited regions and the production of suitable antigens and antibodies is needed to accelerate the development of a rapid serological test.

Interestingly, no significant difference was observed when the aggregates (peak 1) or the monomers (peak 2) were used separately in the ELISA. This finding suggests that aggregates do not hinder the protein’s diagnostic efficacy in an ELISA format. However, the suitability of the produced Gc for other applications, such as lateral flow assays, needs to be evaluated. The produced CCHFV Gc expressed in this study has added application in antibody isolation from single B cells using its avi-tag that can be biotinylated. In a recent report, a recombinant polyprotein bait (rGn/Gc) was used to isolate protective neutralizing antibodies from CCHF-convalescent donors [21]. The resulting monoclonal antibodies mainly targeted some conserved epitopes in Gc. It is not evident from the report if the recombinant Gc used was a monomer, as only SDS-PAGE data are available. Avi-tagged Gc can be biotinylated using various biotinylation kits and the biotin-avidin interaction can be exploited to label CCHFV Gc with various fluorophores. This approach has been successfully used to isolate monoclonal antibodies from convalescent blood donors in several studies [21, 37–40]. Protein aggregation as observed from the SEC can however impact the adequate labeling of proteins with biotin by affecting the accessibility of biotinylation sites. Furthermore, protein aggregation in biotherapeutics has been identified to increase immunogenicity, leading to immune-mediated adverse effects [41]. This could have an impact when the CCHFV Gc is used as a vaccine candidate alone or in combination with a recently reported adenoviral vector vaccine against CCHF [20].

This study highlights the necessity of employing analytical techniques to monitor and limit the formation of protein aggregates to ensure their integrity in downstream applications. Ideally, eliminating aggregates could improve the yield and quality of the Gc antigen. Further research will aim at validating the ELISA using a larger and more diverse panel of samples from various endemic regions to ensure robustness and reliability.

## Conclusion

This study represents a significant advance in CCHFV research by demonstrating the successful expression and purification of the Gc antigen in mammalian 293 F cells using a human insulin leader sequence. The affinity chromatography purification process yielded a protein that shows promise for diagnostic use in ELISA assays and potential for development into lateral flow assays for point-of-care testing, particularly in regions where rapid and accessible diagnostics are desperately needed. The work lays a foundation for further validation studies to assess the performance of the Gc antigen in diverse diagnostic platforms and to investigate its immunogenicity for vaccine development. Additionally, the Gc antigen could be pivotal in isolating neutralizing antibodies, offering therapeutic possibilities for a disease that currently lacks specific treatments. Future efforts will focus on overcoming the challenges of scale-up production to meet the needs for large-scale diagnostics and vaccine manufacturing.

### Electronic supplementary material

Below is the link to the electronic supplementary material.

Supplementary Material 1

## Data Availability

No datasets were generated or analysed during the current study.
