# Supplementary material for: Purification and characterization of soluble recombinant Crimean-Congo hemorrhagic fever virus glycoprotein Gc expressed in mammalian 293F cells
Source: BMC Biotechnol. 2024 Aug 27;24:59. doi: 10.1186/s12896-024-00885-y (PMC11348531; doi:10.1186/s12896-024-00885-y)

**Supplementary Materials**

**Figure S1:** Comparison of GPC and Human insulin leader sequence

**
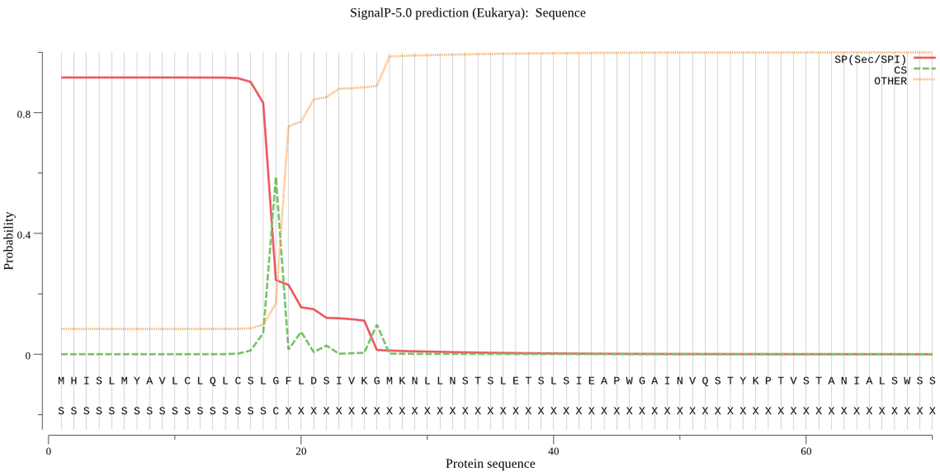
**

CCHFV GPC leader sequence

**Prediction: Signal peptide (Sec/SPI)Cleavage site between pos. 18 and 19: SLG-FL. Probability: 0.5872**

**
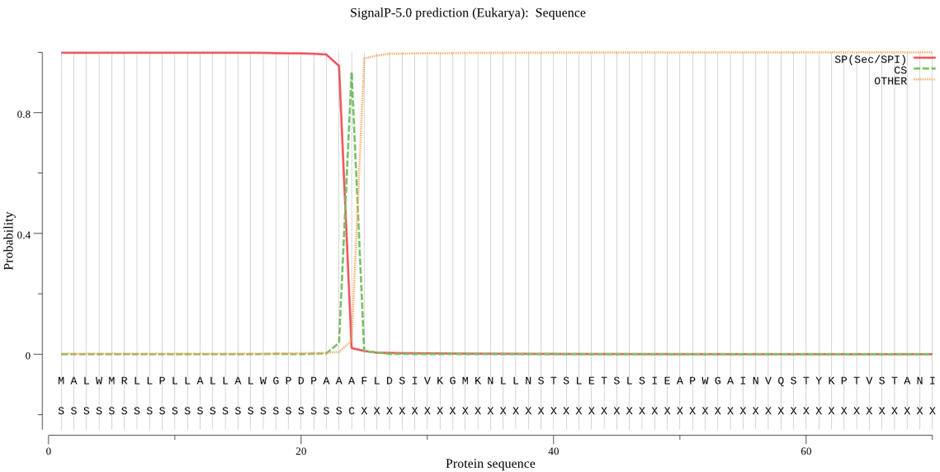
**

Human Insulin leader peptide

**Prediction: Signal peptide (Sec/SPI)Cleavage site between pos. 24 and 25: AAA-FL. Probability: 0.9348**

**Figure S2:** CCHFV Gc structure and estimation of molecular weight

10 20 30 40 50 60
FLDSIVKGMK NLLNSTSLET SLSIEAPWGA INVQSTYKPT VSTANIALSW SSVEHRGNKI

 70 80 90 100 110 120
LVSGRSESIM KLEERTGISW DLGVEDASES KLLTVSVMDL SQMYSPVFEY LSGDRQVEEW

 130 140 150 160 170 180
PKATCTGDCP ERCGCTSSTC LHKEWPHSRN WRCNPTWCWG VGTGCTCCGL DVKDLFTDYM

 190 200 210 220 230 240
FVKWKVEYIK TEAIVCVELT SQERQCSLIE AGTRFNLGPV TITLSEPRNI QQKLPPEIIT

 250 260 270 280 290 300
LHPRIEEGFF DLMHVQKVLS ASTVCKLQSC THGVPGDLQV YHIGNLLKGD KVNGHLIHKI

 310 320 330 340 350 360
EPHFNTSWMS WDGCDLDYYC NMGDWPSCTY TGVTQHNHAS FVNLLNIETD YTKNFHFHSK

 370 380 390 400 410 420
RVTAHGDTPQ LDLKARPTYG AGEITVLVEV ADMELHTKKI EISGLKFASL TCTGCYACSS

 430 440 450 460 470 480
GISCKVRIHV DEPDELTVHV KSDDPDVVAA SSSLMARKLE FGTDSIFKAF SAMPKTSLCF

 490 500 510 520 530 540
YIVEREHCKS CSEEDTKKCV NTKLEQPQSI LIEHKGTIIG KQNSTCTAKT SCWLESVKSF

 550 560 570
FYGLKNMLSG IFGNVGLNDI FEAQKIEWHE HHHHHH

**Theoretical pI/Mw (average) for the user-entered sequence:** Theoretical pI/Mw: 6.03 / 64558.46

**Figure S3:** Predicition of N-glycosylation sites on CCHFV Gc sequence

**Supplementary Table S1:** CCHF positive and negative samples tested by the commercial Crimean-Congo fever virus Mosaic 2 (IgG) kit (EUROIMMUN) and in this study.

| Sample ID | ELISA results |
| --- | --- |
| 66/08 | Positive |
| 67/08 | Positive |
| 69/08 | Positive |
| 6/09 | Positive |
| 30/10 | Positive |
| 51/10 | Positive |
| 52/10 | Positive |
| 53/10 | Positive |
| 6/11 | Positive |
| 31/11 | Positive |
| 51/11 | Positive |
| 54/11 | Positive |
| 41/13 | negative |
| 42/13 | negative |
| 43/13 | negative |
| 45/13 | negative |
| 46/13 | negative |
| 47/13 | negative |
| 49/13 | negative |
| 50/13 | negative |
| 51/13 | negative |
| 52/13 | negative |
| 53/13 | negative |

**Original gels/blots figure 2**

**Figure 2B and 2D**

**
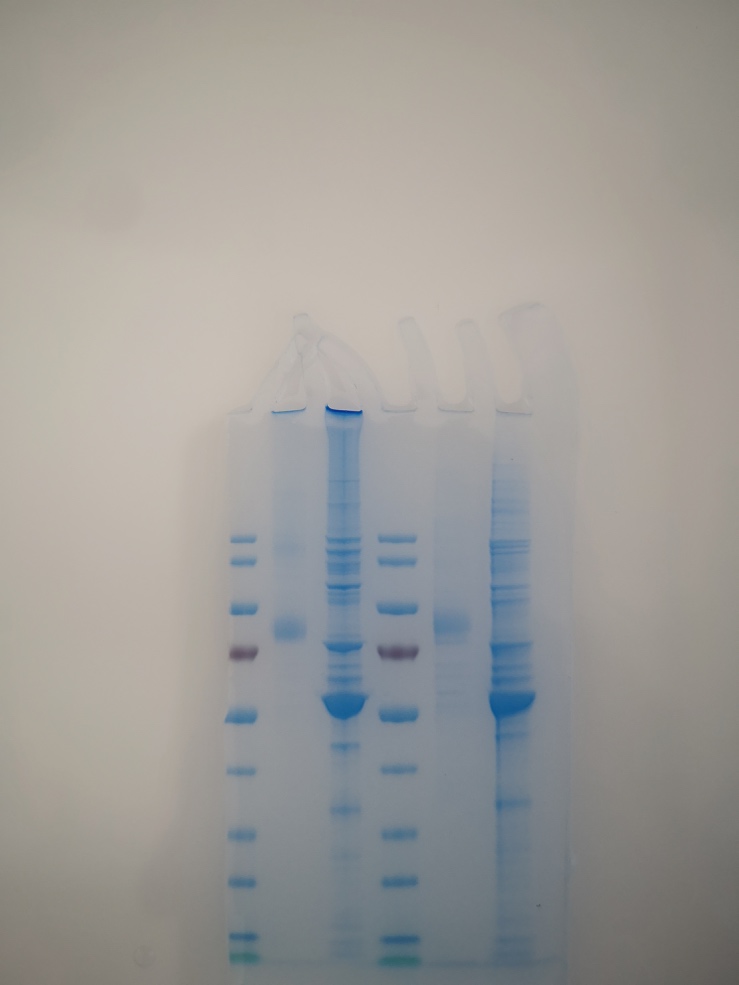
**

**Figure 2C and 2E**


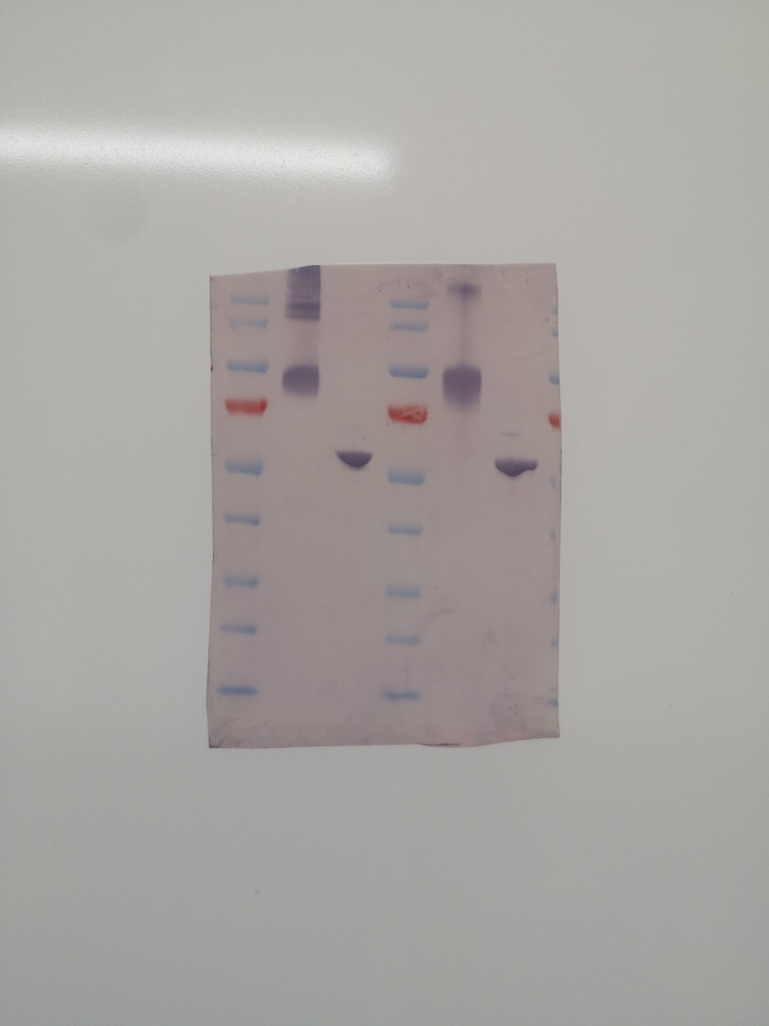


**Figure 2 F: different concentrations of triton X (1%, 0.5% 0.25%)**


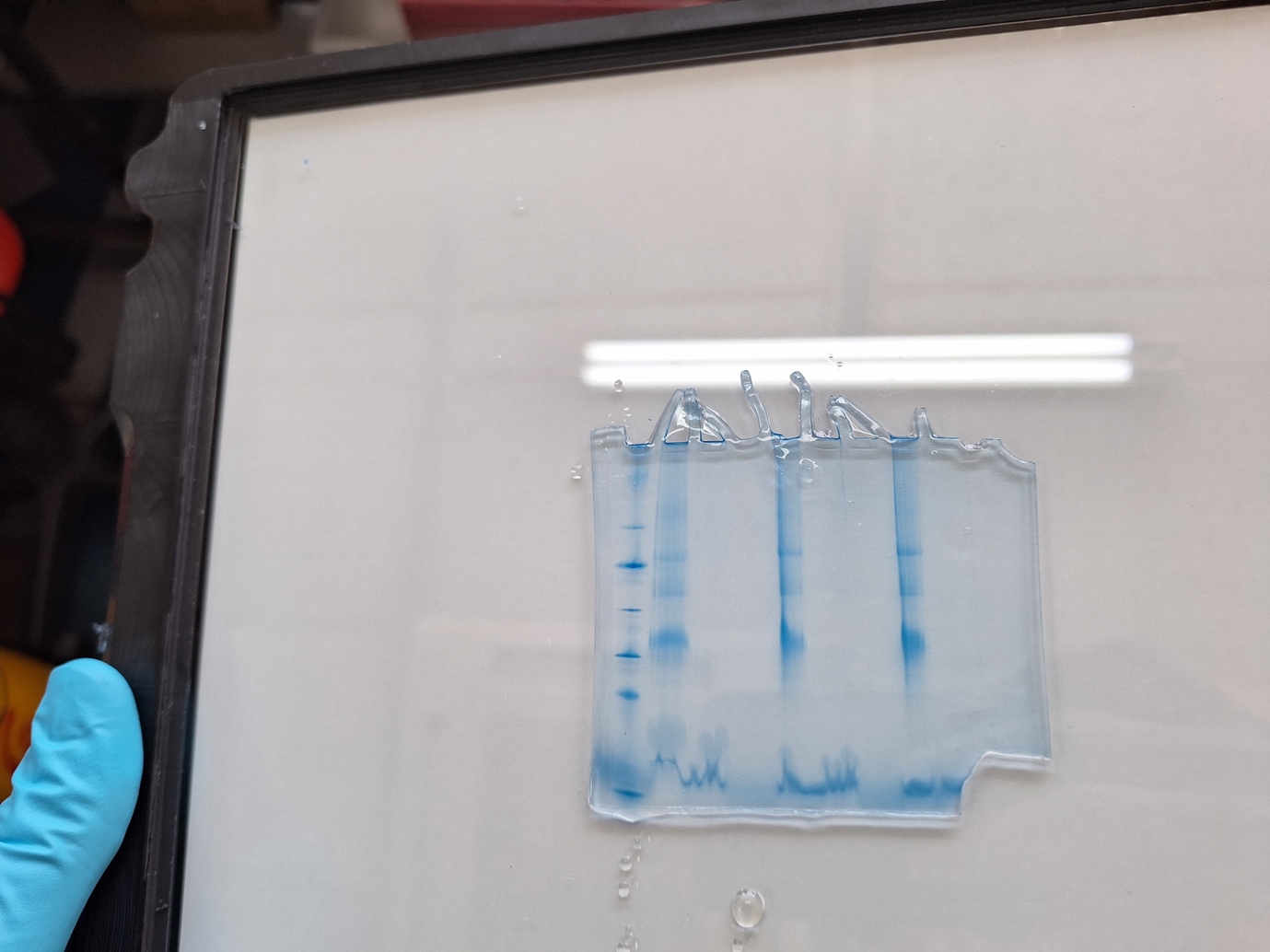


**Original gels/blots figure 3**

**Figure 3 C**

**
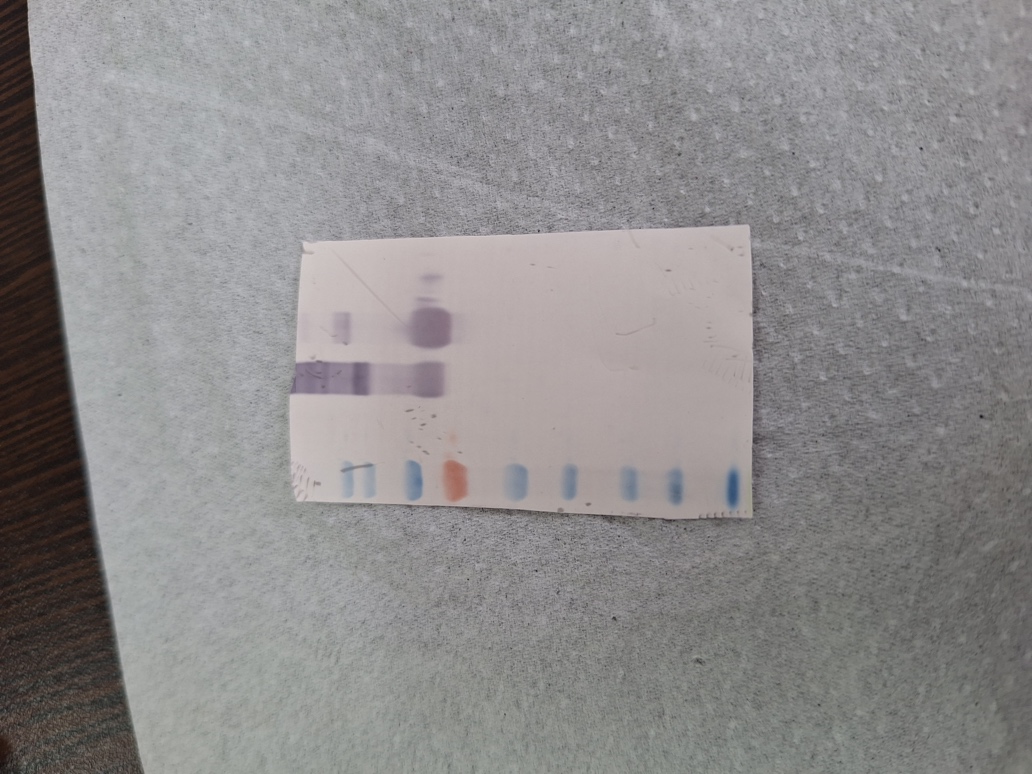
**

**Figure 3B**

**
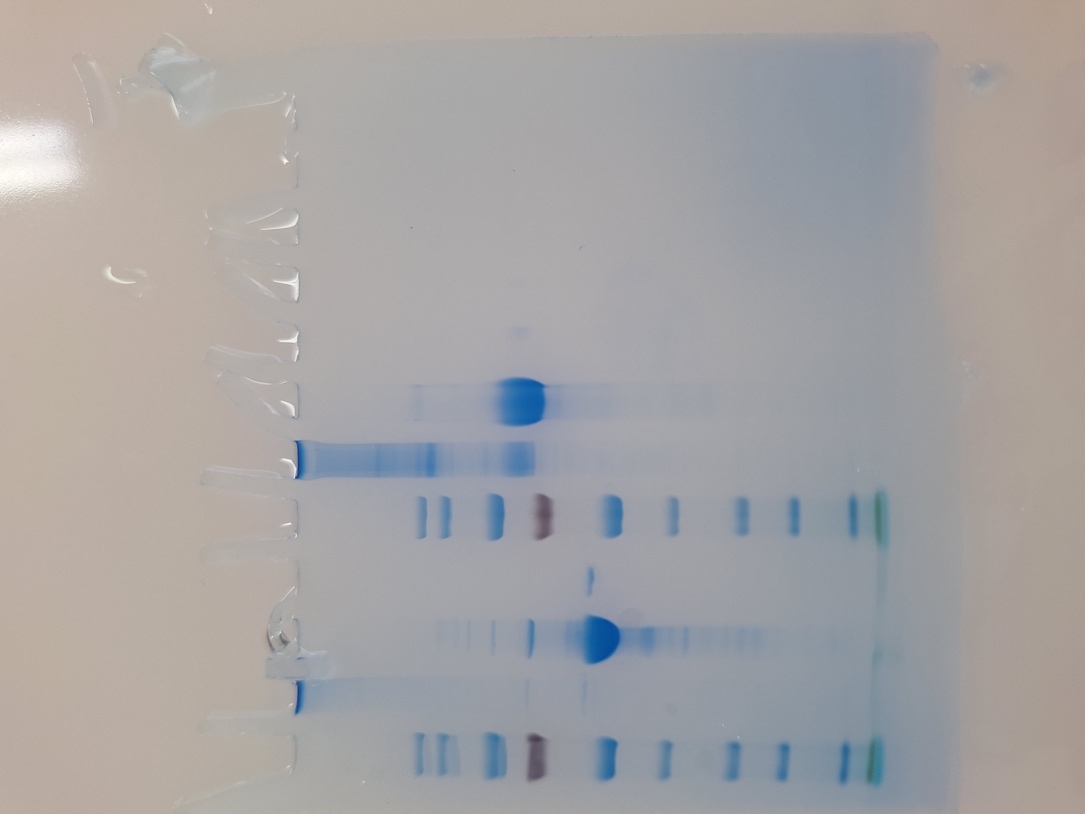
**

**Original gels/blots figure 5**

**Figure 5A**


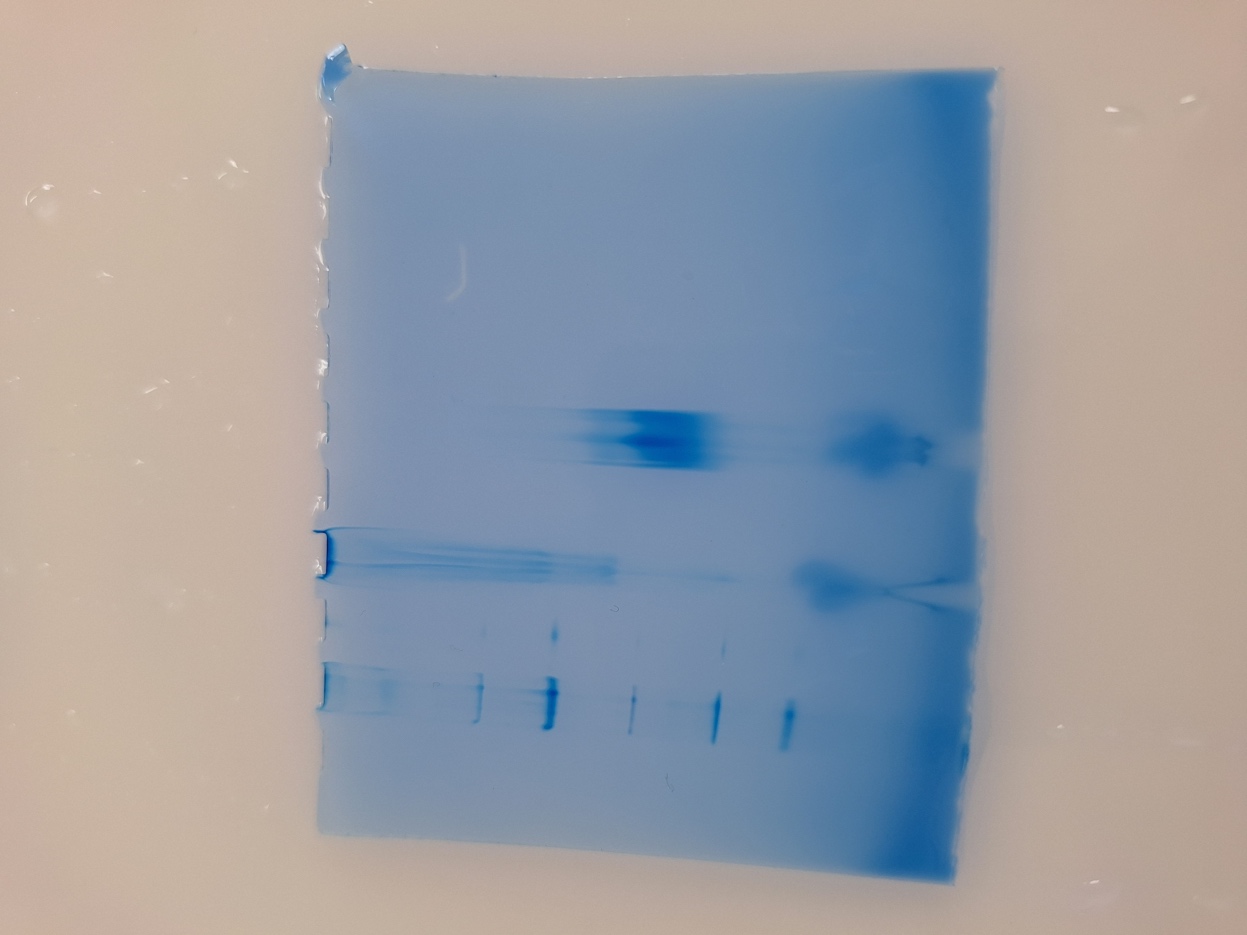

Supplement: Supplementary file 1 — Supplementary Material 1 [file 12896_2024_885_MOESM1_ESM.docx]
